# How Properties of Solid Surfaces Modulate the Nucleation of Gas Hydrate

**DOI:** 10.1038/srep12747

**Published:** 2015-07-31

**Authors:** Dongsheng Bai, Guangjin Chen, Xianren Zhang, Amadeu K. Sum, Wenchuan Wang

**Affiliations:** 1 Department of Chemistry, School of Science, Beijing Technology and Business University, Beijing, 100048, P. R. China; 2 Division of Molecular and Materials Simulation, State Key Laboratory of Organic-Inorganic Composites, Beijing University of Chemical Technology, Beijing, 100029, P. R. China; 3 State Key Laboratory of Heavy Oil Processing, School of Chemical Engineering, China University of Petroleum, Beijing, 102249, P. R. China; 4 Center for Hydrate Research, Department of Chemical & Biological Engineering, Colorado School of Mines, Golden, CO 80401, U.S.A

## Abstract

Molecular dynamics simulations were performed for CO_2_ dissolved in water near silica surfaces to investigate how the hydrophilicity and crystallinity of solid surfaces modulate the local structure of adjacent molecules and the nucleation of CO_2_ hydrates. Our simulations reveal that the hydrophilicity of solid surfaces can change the local structure of water molecules and gas distribution near liquid-solid interfaces, and thus alter the mechanism and dynamics of gas hydrate nucleation. Interestingly, we find that hydrate nucleation tends to occur more easily on relatively less hydrophilic surfaces. Different from surface hydrophilicity, surface crystallinity shows a weak effect on the local structure of adjacent water molecules and on gas hydrate nucleation. At the initial stage of gas hydrate growth, however, the structuring of molecules induced by crystalline surfaces are more ordered than that induced by amorphous solid surfaces.

Clathrate hydrates are ice-like crystalline solid compounds consisting of water (host) molecules and small hydrophobic (guest) molecules, in which guest molecules are enclosed in cages formed by hydrogen bonded water molecules^1^. Three main types of crystalline structure of gas hydrates exist^1^,^2^: sI, sII, and sH. Under natural conditions, gas hydrate is normally formed in porous media, in which solid surfaces play an important role in the formation and dissociation of hydrates^1^,^2^. The effect of solid surfaces on hydrate formation may be attributed to changes in the local structure of water molecules near the liquid-solid interface, thus altering the pathways of hydrate formation. Experimental measurements, for example, show that the formation rate of CH_4_ hydrate in the presence of bentonite surfaces is faster than that in the bulk solution^3^, possibly because the surfaces provide nucleation sites for hydrates to form.

While the mechanism of hydrate formation on different solid surfaces is an attractive and important topic, it is difficult to investigate hydrate nucleation by experimental methods owing to the temporal and spatial resolution limitations of laboratory scale monitoring techniques^4^,^5^,^6^. Alternatively, molecular simulations is a powerful method to provide molecular-level details on hydrate nucleation mechanism. Many molecular simulation studies have focused on the dynamics (formation and dissociation) of hydrates in both bulk two-phase (e.g., vapor-liquid) systems^7^,^8^,^9^,^10^,^11^,^12^ and confined three-phase (e.g., solid-vapor-liquid) systems^13^,^14^. The properties of water near the hydrophilic silica surfaces is different from that in the bulk phase^13^,^14^, and the surfaces can promote the growth of methane hydrate^14^. In our previous studies, we investigated the nucleation process of CO_2_ hydrate induced by hydroxylated SiO_2_ surfaces in a two-phase^15^ and three-phase^16^ systems. We found the existence of silica surfaces can accelerate hydrate nucleation partly because the silica surface stabilized structuring of the adsorbed water.

In nature, gas hydrates can form in different geological environments, for example, in sedimentary rock or unconsolidated clay. Thus, different solid surfaces from crystalline to amorphous and those with hydrophilic to hydrophobic characteristics should be considered in the study of hydrate nucleation. As such, the important fundamental question on how surface properties affect the hydrate formation must be understood.

In this work, a number of solid surfaces with different hydrophilicity and crystallinity are considered to study the nucleation mechanism and dynamics of hydrate formed from those surfaces. We choose the formation of CO_2_ hydrate from silica surfaces for this study. The choice of CO_2_ hydrate comes from the interest in sequestering CO_2_, a greenhouse gas, by ocean storage technologies, such as the direct injection of CO_2_ into the ocean^17^,^18^,^19^,^20^,^21^. In such case, the injection of CO_2_ into sediments could sequester CO_2_ in the hydrate form^1^,^22^,^23^,^24^,^25^, and also create a CO_2_ hydrate layer that would prevent liquid CO_2_ from dissolving into the seawater. The successful application of this approach requires a fundamental understanding of the mechanism of CO_2_ hydrate nucleation in the presence of silica surfaces. The consideration of different types of surfaces (crystalline/amorphous and hydrophilicity) are important since most actual surfaces, including sedimentary rock or unconsolidated clay, are rough and non-uniform in terms of hydrophilicity.

## Results

**Figure 1:**
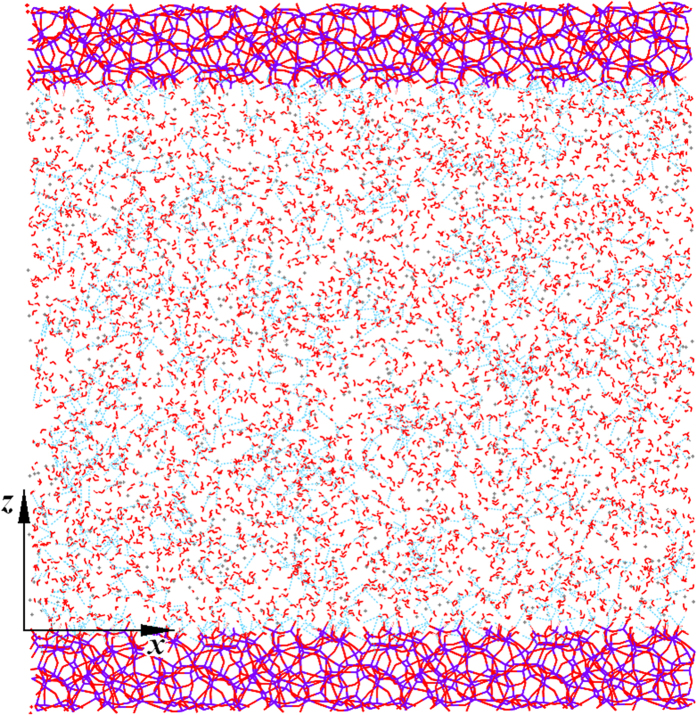
Initial configuration for a typical simulation run. In the figure, the Si and O atoms in the silica are shown as purple and red wires; H_2_O molecules are represented by the stick models in red; C atoms in CO_2_ molecules are denoted by gray dots while O atoms are omitted for clarity. Hydrogen bonds are described as blue dashed lines.

**Figure 2:**
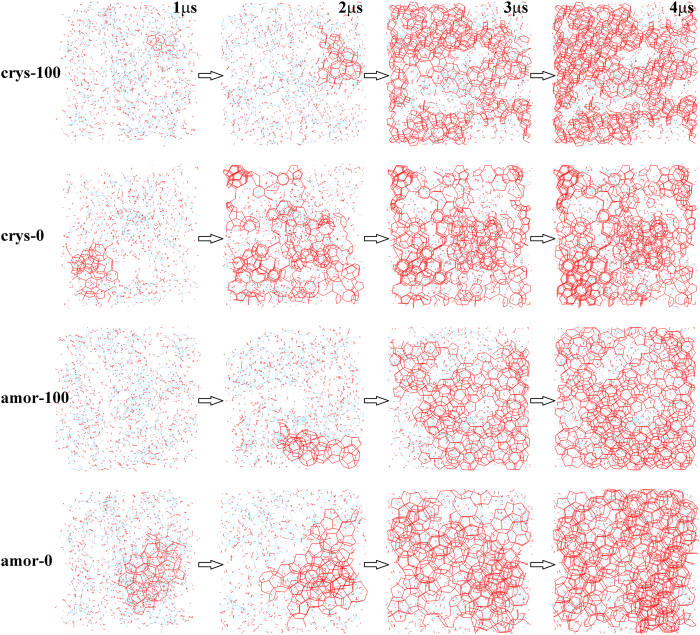
Typical snapshots for several systems during the hydrate formation process. In the figure, only region C of the systems (denoted in Fig. 4) are shown in *xy*-plane. For clarity, CO_2_ molecules are omitted. Color code for the snapshots is the same as in Fig. 1, and the hydrate cages are shown with red wire-frame bonds.

**Figure 3:**
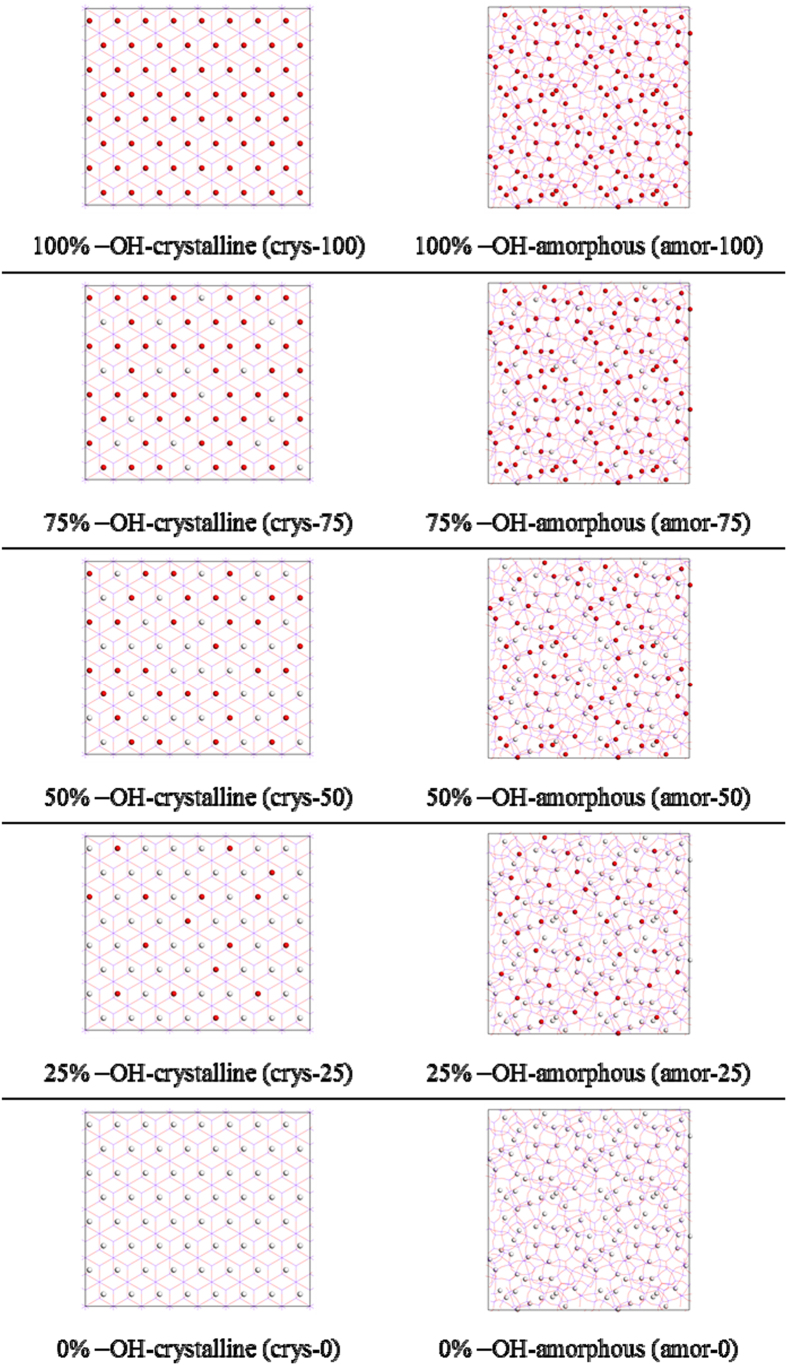
Top view of various silica surfaces studied in this work. The red and white spheres represent –OH and –H groups on silica surfaces, respectively. In those systems, the type of –OH or –H group is assigned randomly while keeping the percentage of –OH groups to a given value.

Series of microsecond scale molecular dynamics simulations were performed to investigate the nucleation mechanisms of hydrate with silica surfaces have different properties. A typical initial configuration is shown in Fig. 1, and the hydrate formation processes for several systems are shown in Fig. 2. To simplify the description, systems with different silica surfaces are marked as crys-*n* or amor-*n*, for which the ‘crys’ or ‘amor’ corresponds to a crystalline or amorphous solid surface, and the number of *n* indicates the percentage of –OH groups saturating the surface (see Fig. 3 in Methods section for details). Therefore, from the crys-100 to the crys-0 systems, the –OH groups saturating the crystalline surfaces are increasingly changed into –H groups, decreasing the solid surface hydrophilicity.

### Effect of solid surfaces hydrophilicity

**Figure 4:**
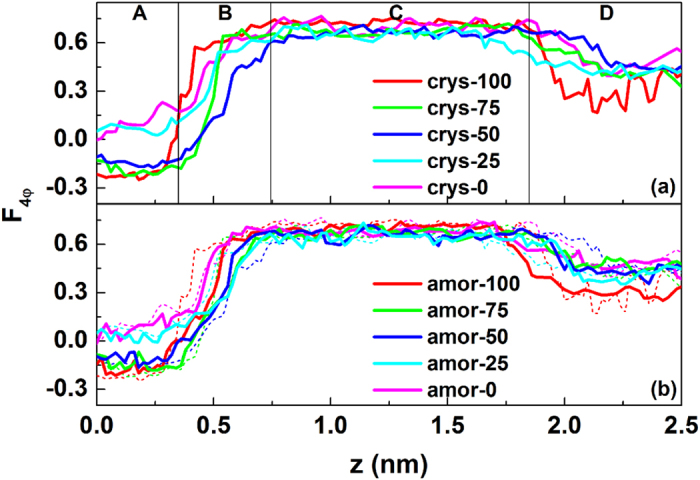
The distributions of 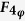 along *z* direction in the final configurations for systems with (**a**) crystalline silica surfaces and (**b**) amorphous surfaces. In the figure, only the lower half part of the system is shown, and regions A, B, C, and D, divided based on the difference in the local structuring of water molecules^15^, are introduced to describe the local water structure as a function of the distance from silica surfaces. As a comparison, the curves in upper panel are also shown as dashed lines in the same color in the lower panel.

Hydrate nucleation mechanism was first studied for crystalline surfaces of varying hydrophilicity. A four-body structural order parameter 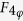
26,27 was used to monitor the structural change of water molecules. The order parameter is a function of the torsional angle between oxygen atoms within 3 Å and the outermost hydrogen atoms in the H_2_O-H_2_O pair, which is defined as 
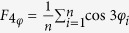
. Here, *φ*_*i*_ is the torsional angle of the *i*th H_2_O-H_2_O pair, and *n* is the total number of H_2_O-H_2_O pairs. Note that for the bulk system, the values of 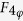 for hydrate, liquid water and ice are 0.7, −0.04 and –0.4, respectively^28^. Since the simulation systems in this study are not a bulk water system, the structure of water close to the surface might be distorted due to the existence of silica. Hence, we note that a solid-like structure of water with a minus value of 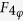 will be classified into an ice-like structure. Figure 4a shows the obtained values of 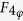 for various systems with different surface hydrophilicity. In general, CO_2_ hydrate was formed within 4 μs for all the silica surfaces studied, indicating the presence of hydrophilic surfaces prompts hydrate nucleation.

In our previous study^15^, we considered a super-saturated CO_2_ solution system with 100% –OH groups and crystalline silica surface (denoted as crys-100, see Fig. 3), and it shows a three-step nucleation mechanism: an ice-like layer closest to the silica surface formed first, followed by an intermediate thin layer subsequently formed to compensate for the structure mismatch between the ice-like layer and the final stable CO_2_ hydrate, and finally a CO_2_ hydrate layer generated from the intermediate structure acting as a nucleation zone. In this work, although the concentration of CO_2_ is decreased, the three-step nucleation mechanism is also existed for crys-100 system. However, as we will discussed below, the nucleation mechanism will be changed when hydrophilicity of the surface changes.

For the solid surfaces with weak hydrophilicity (crys-25 and crys-0), the amorphous ice-like layer closest to the silica surface vanishes, only a single liquid-like intermediate layer is formed directly on the surface, and as a result the nucleation mechanism changes. The diffusion coefficients of water molecules in the region closest to the solid surface are calculated based on the mean square displacement, and the results are listed in Table 1. One can see clearly that for the system of crys/amor-100, 75 and 50, the diffusion coefficients are small enough, indicating a solid-like layer formed. For the system of crys/amor-25 and 0, however, the diffusion coefficients increased by one order of magnitude, indicating a liquid-like layer close to the solid surface. Figure 4a also shows that for weakly hydrophilic surfaces, the 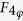 in region A is close to that for liquid water, indicating that the CO_2_ hydrate directly nucleates through an intermediate (liquid-like) water layer coating on the surfaces, rather than from an ice-like water layer as seen for strongly hydrophilic surfaces (crys-100). As a result, the three-step nucleation mechanism^15^ changes into a two-step nucleation mechanism.

To determine the reason for the variation of nucleation mechanism as a function of surface hydrophilicity, we investigated the local water structure near the silica surfaces. The results show that the water layer close to the solid surfaces becomes less structured when the surface hydrophilicity decreases. For example, for the crys-0 system (the solid surfaces with the lowest degree of hydrophilicity), the value of 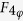 close to the silica surface increases to 0.05, significantly deviating from −0.2 for the crys-100 interface (Fig. 4a). This observation again proves that for the solid surfaces with low hydrophilicity the amorphous ice-like interfacial layer vanishes, and a liquid-like interfacial water layer appears.

An interesting observation found is that the amorphous ice-like water layer close to the silica surfaces can persist even for the case with 50% –OH groups. As is shown in Fig. 4a for the crys-75 and crys-50 systems, the 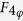 value for the interfacial layer (region A) fluctuates around −0.2, similar to that in the crys-100 system. However, for the crys-25 system, the small number of –OH groups is insufficient to stabilize the ice-like structure of the water layer, and as a result, the ice-like layer disappears. This is confirmed by the observation that near the silica surface, both the value of 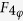 increases to 0.05 (Fig. 4a), and the diffusion coefficient increases to ~10^−9^ m^2^/s, similarly to that for the crys-0 system.

**Figure 5:**
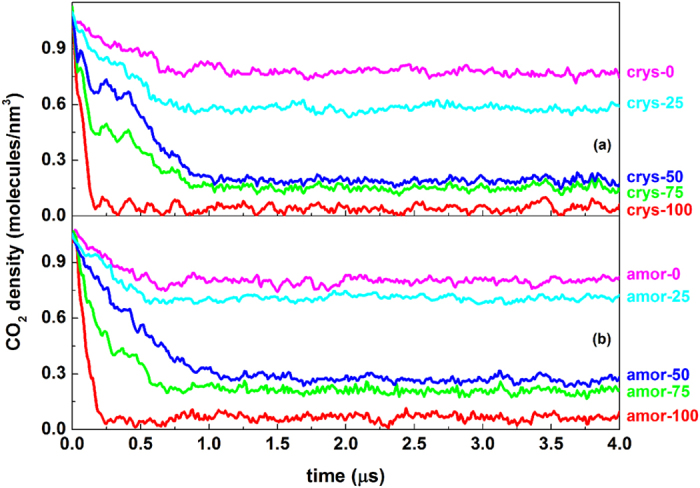
Evolution of CO_2_ number densities in a region within 0.75 nm from silica surfaces. For all the systems, the composition of fluid phase is chosen with a CO_2_ number density of ~1.04 molecules/nm^3^.

Weakening surface hydrophilicity not only changes the local structure of water molecules at the interface, but also weakens the tendency for CO_2_ molecules to leave from the solid surface. Figure 5 gives the evolution of CO_2_ density in the region close to the silica surfaces (within a distance of 0.75 nm). The figure shows that the final CO_2_ density near a silica surface depends strongly on the surface hydrophilicity: the more hydrophilic the surface is, the lesser the number of CO_2_ molecules are remained. For weakly hydrophilic surfaces, a relatively large amount of CO_2_ molecules are remained on the surfaces, and inevitably, the existence of CO_2_ molecules near the silica surface would affect the local structure of the water layer closest to the surface. The existence of CO_2_ in the water layer can hinder the full development of the hydrogen-bond network, and should be considered as another reason for the disappearance of amorphous ice-like layer (region A), especially for the system with surfaces weakly hydrophilic.

The effect of surface hydrophilicity on the distribution of CO_2_ molecules is consistent with that of local structure of the water layer. From the crys-75 to crys-50 systems, the corresponding CO_2_ densities of those systems are just slightly increased compared with the crys-100 system, whereas the hydrophilicity of the silica surface is significantly decreased (Fig. 5). For the crys-25 and crys-0 systems, however, there are less –OH groups to hold the water molecules, and instead the surface can adsorbed more CO_2_ molecules. As such, one could suggest that the –H-modified silica surface can be considered CO_2_-philic.

Moreover, the calculation results of the density of water molecules in region A (Table 1) show that the amorphous ice-like layer is a low-density “ice”. With the weakening of the surface hydrophilicity, one can see that the density of water is decreased. This is partly because the adsorption capacity of solid surfaces decreased when –OH groups are changed to –H groups (see Discussion section for details), and partly because the amount of CO_2_ molecules remained within the layer becomes larger.

**Figure 6:**
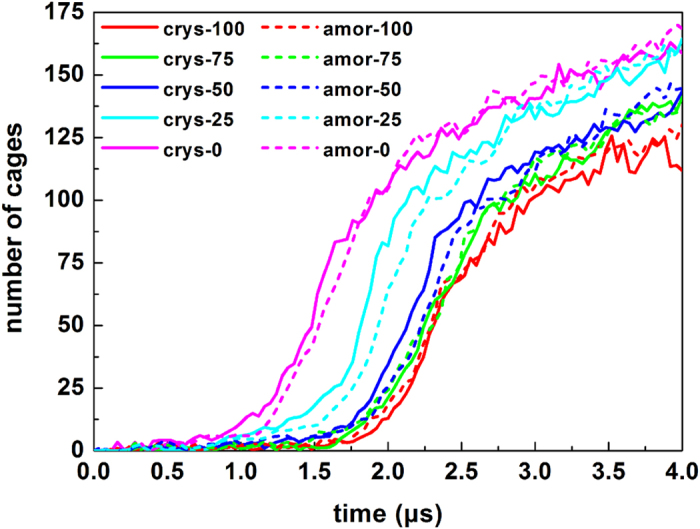
Evolution of the number of cages during different nucleation processes.

The presence of hydrophilic surfaces also affects the dynamics of hydrate nucleation. By using the ring perception^29^ and the cage identification^30^ algorithms, we calculated the time evolution of the number of hydrate cages, as shown in Fig. 6. From the figure it is surprising to find that the induction time for the crystal nucleation is reduced when less –OH groups exist on the silica surface. In other words, CO_2_ hydrates can be more easily formed from less hydrophilic solid surfaces.

### Effect of surface crystallinity

The study of the effect of the silica surfaces crystallinity on hydrate formation process is started by comparing the crystalline and amorphous surfaces. Figure 4b shows the values of 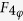 for several crystalline and amorphous surfaces. It is clearly show that 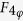 for water molecules near amorphous and crystalline surfaces have about the same value as long as the surface hydrophilicity is the same. As such, the water and hydrate structures near silica surfaces mainly depend on the surface hydrophilicity (i.e., number of –OH groups), rather than surface crystallinity. We note that the roughness introduced in our model amorphous surface is in the atomic scale, different from the larger surface roughness on amorphous surface, such as pits, wedges, and pore. Sear and coworkers^31^ demonstrated that the roughness in larger scale would cause a stronger influence on the liquid-solid nucleation. However, simulation on the amorphous surfaces gave the same trend on the effect of surface crystallinity, and showed again that the effect of the atomic scale roughness on hydrate nucleation is rather weak.

On the other hand, in comparison with the systems with crystalline silica surfaces (Fig. 5a), the CO_2_ density on amorphous silica surface is kept slightly higher (Fig. 5b). More CO_2_ molecules remained on amorphous surfaces in turn perturb the formation of the hydrogen bond network, and consequently, more free water molecules are present in the adjacent water layer.

Figure 6 shows that the induction time for the hydrate nucleation is nearly independent on surface crystallinity because the local water structure is weakly affected by the surface crystalline. The growth rate of the hydrate after nucleation is also roughly the same for different systems (see Fig. 6). This is because the subsequent hydrate growth after nucleation is far from silica surfaces, and any surface effect is shielded by the initial hydrate layer formed. As a result, the growth rate of hydrate is mainly controlled by temperature, pressure and local concentration of CO_2_, rather than the properties of solid surfaces.

**Figure 7:**
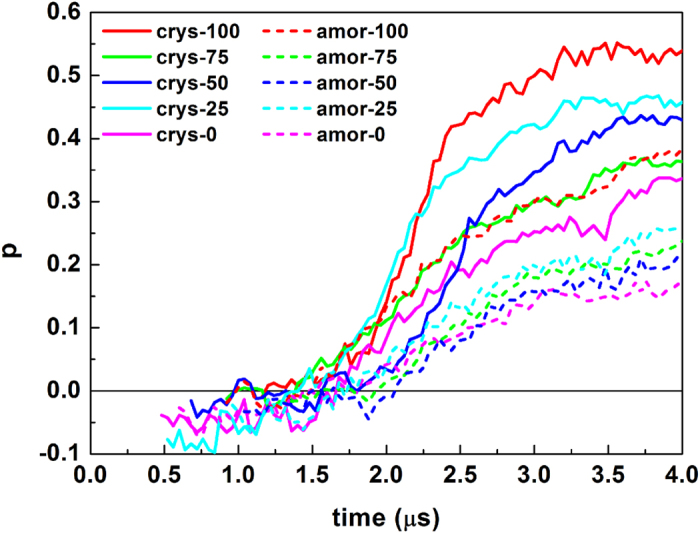
Evolution of the order parameter *p*. Note that *p* = 1 corresponds to a pure sI crystal, while *p *= −1 represents a pure sII crystal.

**Figure 8:**
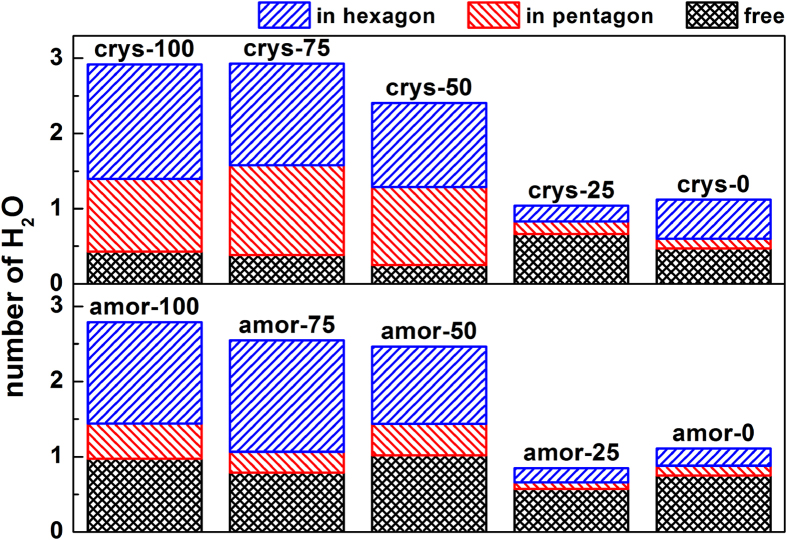
The local structure of water molecules closest to silica surfaces. In the figure, the number of H_2_O adsorbed denotes the average amount of the adsorbed water molecules per –OH or –H group. The adsorbed water molecules may come from hexagonal water rings, pentagonal rings, and free ones. Note that free water molecules here are the water molecules that do not belong to hexagons and pentagons, and they might appear as dimers, trimers, tretamers, or even rarely observed heptamers.

Although different systems have similar nucleation time and growth rate as long as the hydrophilicity of these surfaces is similar (Fig. 6), the degree of order for those hydrate crystals does depend on the surface crystallinity. Figure 7 shows the time evolution of the order parameter 
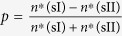
,^16^ which was designed to describe the order of the hydrate crystal. Figure 7 shows that all hydrate crystals are dominated by the sI-like component, as indicated by the positive value of *p*. However, it is found that the hydrate induced by a crystalline silica surface is more ordered than by an amorphous one, even though the growth rates of those hydrates are similar. This observation follows from the fact that amorphous surfaces lead to a more disordered hydrogen bond network (Fig. 8), and hence the intermediate layer contains more free water molecules, which may reduce the order of the hydrate crystals.

## Discussion

The change of nucleation mechanism (i.e. three-step nucleation or two-step nucleation) is mainly caused by the local structure of water close to the solid surface. To address the dependence of local water structure on surface hydrophilicity, we further analyzed in detail the structure of water molecules closest to the silica surface. Figure 8 shows that for the crys-100 system, an –OH group can adsorb on average about 2.8 water molecules, and most of these adsorbed water molecules are part of hexagonal or pentagonal water rings (identified by the ring perception algorithm^29^). However, we note that although this layer contains several hexagonal rings, it not contains single-crystal motifs of any ice lattice; it is an amorphous layer with solid state. This is also the reason that the adsorbed water layer is called amorphous “ice-like”.

When the hydrophilicity of the silica surfaces is continuously weakened from crys-100 to crys-0 systems, the average number of water molecules hydrogen bonded with silica (per –OH or –H group) decreases from ~3 to ~1, in which the fraction of water molecules forming 5- and 6-membered water rings decreases accordingly and that of free water molecules increases (Fig. 8). For example, for the crys-0 system (the least hydrophilic system), the number of water molecules hydrogen bonding to the silica surface significantly decreases. Only 1.1 water molecules are adsorbed to each –H group, and the percentage of free water molecules can be as large as 42% (Fig. 8). In this case, therefore, the mobility of water molecules increases, and consequently the ice-like water layer disappears (region A in Fig. 4a).

Consistent with the change of 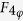 as a function. of surface hydrophilicity, the structure of the water layer near the surface is still relatively unchanged even though the number of –OH groups decreases to less than 50%. This is because even though the number of water molecules hydrogen bonding with the silica surface directly decrease with the decrease of –OH groups, the structure can still be stabilized alternatively by forming hydrogen bonds with its neighboring water molecules which are stabilized by hydrogen bonding with the –OH or –H groups. The MD simulation study on confined water^32^ indicated that the confining of water molecules on translation is stronger than that on rotation. It might be an explanation for the issue that why the local structure of water in the region close to the surface in the crys-50 system is similar to that in crys-100 system, rather than that in crys-0 system. This is because the rotation of water molecules can hold the network of hydrogen bonds undestroyed. But when the –OH groups decreases to 25% (crys-25) or less (crys-0), the ability of the silica surface to form hydrogen bond with water molecules significantly decreases so that the surface cannot stabilize the ice-like structure in the interfacial layer. Thus, the percentage of free water molecules significantly increases (Fig. 8). Similar results are obtained in confined silica-water binary system^33^. They found that the structure of water layer closest to the silica is more ordered and highly confined when silica surface has larger –OH density, i.e. more hydrophilic. In general, the surface hydrophilicity can change the local water structure, determining the presence or absence of the amorphous ice-like layer and inducing a change in the nucleation mechanism.

**Figure 9:**
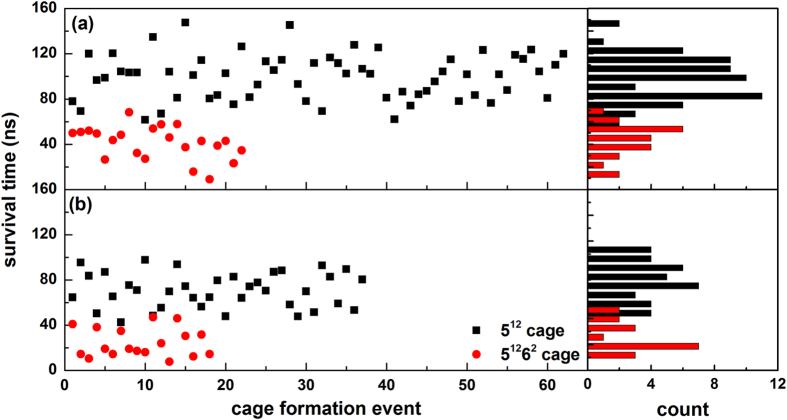
Survival time of different kinds of cages during nucleation stages for (**a**) crys-100 and (**b**) crys-0 systems.

Figure 9 gives the lifetime of 5^12^ and 5^12^6^2^ cages during the nucleation stages. The figure shows that hydrophilic surfaces prolong the lifetime of cages, indicating that the hydrophilic surfaces act as stabilizer for the adjacent cages, as suggested in our previous simulation results^16^. Although a cage hydrogen bonding with solid surfaces indeed increases its lifetime, a larger inaccessible region for hydrate cages near the solid surface, however, is existed for the cages formed on strong hydrophilic surfaces. We ascribe it to the structural mismatch between the hydrate lattice and the local structure of adjacent water layer, which leads to an increase of nucleation time (Fig. 6) and an ice-like water layer is formed firstly on strong hydrophilic surfaces.

The effect of structural mismatch on hydrate nucleation depends on the surface hydrophilicity. For strong hydrophilic surfaces, the structural mismatch between the hydrate lattice and amorphous ice-like layer inhibits the adsorbed hydrate cages growth into fully developed critical nucleus, and hence an intermediate layer has to form to bridge the structure mismatch. On the other hand, in the ice-like layer, the mobility of water molecules is strongly restricted by the silica surfaces, which also inhibits the hydrate nucleation and slows down its dynamics. But for weak hydrophilic surfaces, a liquid-like water layer closest to surfaces appears instead, and the restrictions on the adsorbed water molecules are substantially weakened. This can be confirmed from the diffusion coefficients of water listed in Table 1, which indicating that less hydrophilic surfaces pose less restriction in the mobility of the water molecules, and in consequence, accelerate the nucleation process. We should note that the diffusion coefficients of the ice-like or liquid-like layer close to the surface are heterogeneous: the diffusivity of water molecules in the *xy*-direction is stronger than that in the *z*-direction with respect to the solid surfaces. The anisotropy of water in confined space is a common phenomenon, and similar results are obtained for water between graphite surfaces^34^. In general, the hydrate nucleation tends to occur more easily on less hydrophilic silica surfaces, partly because of the stabilization effect of silica on hydrate cages, and partly because of the availability of free or less-constrained water molecules which are needed for the formation of hydrate cages. Moreover, the diffusion coefficients of the hydrate crystals formed in region C are in the range of 0.18 ~ 0.33 × 10^−9^ m^2^/s for all of the systems, showing a solid feature.

The structure of water molecules at solid-liquid interfaces was also analyzed in detail, as shown in Fig. 8. In comparison with crystalline surfaces, amorphous surfaces induce an increase in the amount of adjacent free water molecules as well as a decrease in pentagonal water rings, while the amount of hexagonal water rings remains unchanged. This is partly due to the amorphous silica surfaces, as surface roughness induces a stronger structural perturbation on adjacent water rings and cages, leading to a more disordered hydrogen bond network. However, the total number of water molecules hydrogen bonding to –OH or –H groups does not significantly change. Consistently, Fig. 4b shows that there is no significant difference between 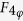 for water molecules near crystalline and amorphous surfaces. In general, our simulations demonstrate that enhanced structural perturbation of amorphous surfaces on hydrate rings and cages leads to a slightly disordered hydrogen bond network when the surface hydrophilicity is similar.

The molar fraction of CO_2_ in water we used is 0.042 in our simulation systems, which is a slightly supersaturated solution (see Method section for details). Since that some of the CO_2_ molecules are remained near the solid surface, the effective degree of supersaturation in different systems will be different, and it is another important factor to affect the hydrate formation rate and the mechanism. At ~0.8 ms for all of the systems, the expulsion of CO_2_ molecules from the region close to the silica surfaces is finished (Fig. 5) and it is ready to start the formation of hydrate (Fig. 6). Hence, we calculated the CO_2_ density in the border region (region A and B) and the central region (region C and D) at 0.8 ms, respectively. Take crys-0 and crys-100 systems for example, the density in border region is 0.775 molecules/nm^3^ for crys-0 system and 0.042 molecules/nm^3^ for another, with a difference of 18.45 times (0.775 divided by 0.042). The density in central region, however, is 1.15 and 1.47 molecules/nm^3^ for the two systems respectively, only have a difference of 1.28 times. Compared with the former, the density difference of CO_2_ in central region between different systems is negligible. Therefore, we point out that the obvious difference in the induction time for hydrate nucleation occurred near the surface (Fig. 6) is owing to both the local structure of water and the density of CO_2_ are significantly different for systems with different silica surfaces. However, the hydrate growth is mainly occurred in the central region, and the rate for all of the systems is roughly the same. This is caused by the similar CO_2_ density in central region and the weakened surface effect. We note that although it is a superstaturated system, the main factor to affect the hydrate nucleating is the properties of water and CO_2_ close to the solid surface, rather than that in the central region of the system. Since the CO_2_ molecules are all expulsed to some extent in different systems, the local degree of supersaturation of CO_2_ in border region well be further reduced. So, the main results we obtained can reflect the real situation of the system in a certain extent. In real system, perhaps the local structuring of water and the expulsing of CO_2_ near the surface become less obvious owing to the low CO_2_ concentration, but the nucleation mechanism will not change.

In summary, we performed microsecond MD simulations to investigate how the presence of hydrophilic silica surfaces modulates the hydrate nucleation. The hydrophilicity of solid surfaces can change the local structure of adjacent water layers: the nucleation mechanism varies from three steps into two steps when surface hydrophilicity becomes weak. It also affects the nucleation dynamics of the hydrate formation: the induction time for nucleation is reduced when the surface is less hydrophilic. While for strong hydrophilic surfaces, the structure mismatch between the hydrate lattice and amorphous ice-like layer inhibits the nucleation and then the nucleation dynamics is slowed down. The crystallinity of solid surfaces affects the hydrate formation process in a rather weak manner. The hydrates induced by crystalline silica are more ordered than by amorphous silica. Owing to the fact that hydrate growth after nucleation is far from the silica surface, the growth rates of the hydrate seem to be independent on both surface hydrophilicity and surface crystallinity.

## Methods

Molecular dynamics simulations were performed by using LAMMPS^35^. The simulations were implemented in a two-phase system that contains a pair of silica layers (solid phase) and a mixed H_2_O/CO_2_ fluid (liquid phase) with 200 CO_2_ and 4600 H_2_O molecules, as is shown in Fig. 1. In the initial configurations, the CO_2_ and H_2_O molecules were randomly placed in a simulation box of 6.22 nm × 6.22 nm × 4.98 nm (roughly equals to 5 × 5 × 4 unit cells of sI hydrate). At the top and bottom of the simulation box, two silica layers were added to represent a slit pore of sedimentary rock. Due to the lack of the solubility of CO_2_ solution within a confined space in our simulation conditions, we extrapolated the solubility of bulk CO_2_ solution to our conditions based on the experimental data^36^,^37^,^38^, which is ~0.037 of CO_2_ molar fraction in water. In our simulation systems, the molar fraction of CO_2_ is 0.042, showing a supersaturated solution. Considering the confining effect, we estimate that the degree of supersaturation of CO_2_ used in the simulations is not higher than 4 times of the real system at the same conditions. In all of the simulations, the positions of SiO_2_ molecules were fixed and periodic boundary conditions were imposed in all three Cartesian directions.

The CO_2_ molecules were represented by the EPM2 model^39^, which has three Lennard-Jones sites with charges centered at each atom and with rigid bond lengths but a harmonic bond angle. The H_2_O molecules were described by the extensively used TIP4P model^40^, in which the rigidity of H_2_O molecules was restricted by the SHAKE algorithm^41^. The silanols/silanes model^42^ was adopted for the SiO_2_ layers. The Lennard-Jones interaction parameters for molecules are reported elsewhere^15^. Note that the force fields used were successfully applied to study the nucleation of CO_2_ hydrates in both two-phase^15^ and three-phase systems^16^. A cutoff radius of 12.0 Å was employed for the short-ranged interactions, and the PPPM algorithm^35^ was used for long-ranged electrostatic interactions. Constant temperature and pressure simulations were maintained at 265 K and 150 bar, respectively, with the Nosé-Hoover algorithm^43^,^44^,^45^. According to experimental phase diagram^1^, under the conditions the hydrate phase is thermodynamic stable. The Newtonian equations of motion were integrated based on the velocity Verlet algorithm^46^ with a time step of 2 fs.

In order to investigate the effect of surface properties on the hydrate nucleation process, we introduced several model solid surfaces with different hydrophilicity and crystallinity, as summarized in Fig. 3. The crystalline silica layers were taken from the (1 1 1) plane of the *β*-cristobalite^47^, while the amorphous silica layers with a roughness down to atomic scale were built by using in total 1060 silicon and 2120 oxygen atoms with a density closing to the experimental value of 2.2 g/cm^3^. For different model SiO_2_ surfaces, dangling bonds on the surfaces were saturated by –OH or –H groups. The –OH group has no rotational freedom around the Si–O bond. Obviously, a silica surface saturated by an –OH group alone (hydroxylated model) is more hydrophilic than that saturated by an –H group (hydrogenized model) because an –OH group covering the silica surface can be associated in hydrogen bonds with three other water molecules. While an –H group can hydrogen bond to a single water molecule at most.

To generate different surface hydrophilicity, the usual method controlling the surface density of the –OH group is to select two locations on the (1 1 1) plane of the *β*-cristobalite^33^ or to select different crystallographic faces of the crystal. When using this method to change the hydrophilicity of solid surfaces, however, the surface structure is also changed. To separate the effects of surface hydrophilicity and surface structure, we introduced another method in which a number of dangling bonds on silica surface are chosen randomly to be saturated with –H groups, to control the surface hydrophilicity. Hence, in this method the variation of the hydrophobicity of a model silica surface does not necessarily change the surface structure.

A typical MD simulation was divided into two steps. First, an *NpT* relaxation process of 2 ns was performed at 265 K and 150 bar to eliminate the effect of the initial configuration. Within the first 1 ns, the oxygen atoms of water molecules were fixed to minimize the total dipole moment of water molecules; then the restriction on the oxygen atoms was gradually removed and the system continued to relax for another 1 ns. A typical configuration after the relaxation process is shown in Fig. 1. The configuration is used as the initial configuration of next step. In the second step, hydrate nucleation and growth simulations were performed at the same *NpT* conditions to 4 ms in total time.

## Additional Information

**How to cite this article**: Bai, D. *et al.* How Properties of Solid Surfaces Modulate the Nucleation of Gas Hydrate. *Sci. Rep.* **5**, 12747; doi: 10.1038/srep12747 (2015).

## Figures and Tables

**Table 1:** The 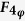 order parameter, diffusion coefficient and density of water molecules in a region within 0.75 nm from silica surface.

	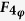	***D*_*xy*_ (10^−9^ m^2^/s)**	***D*_*z*_ (10^−9^ m^2^/s)**	***ρ* (/nm^3^)^a^**
crys-100	−0.22	0.41	0.11	27.24
crys-75	−0.18	0.51	0.097	27.38
crys-50	−0.15	0.70	0.14	26.51
crys-25	0.08	6.33	0.26	15.87
crys-0	0.05	6.82	0.22	13.15
amor-100	−0.20	0.46	0.12	28.33
amor-75	−0.15	0.50	0.10	28.16
amor-50	−0.14	0.73	0.13	27.94
amor-25	0.04	7.29	0.21	18.87
amor-0	0.10	7.51	0.21	17.01

^a^For perfect sI gas hydrate, the calculated density of water is ~23.84 molecules/nm^3^ (46 water molecules in one unit cell which has lattice length of 1.245 nm), while for liquid water, the value is ~33.43 molecules/nm^3^ (1 g/cm^3^).

## References

[b1] Sloan E. D. & Koh C. A. Clathrate Hydrates of Natural Gases, 3rd ed. (CRC Press: Boca Raton, FL, 2008).

[b2] Buffett B. A. Clathrate hydrates. Annu. Rev. Earth Planet. Sci. 28, 477–507 (2000).

[b3] Yan L., Chen G., Pang W. & Liu J. Experimental and modeling study on hydrate formation in wet activated carbon. J. Phys. Chem. B 109, 6025–6030 (2005). 16851658 10.1021/jp045679y

[b4] Shen Y. R. & Ostroverkhov V. Sum-frequency vibrational spectroscopy on water interfaces: polar orientation of water molecules at interfaces. Chem. Rev. 106, 1140–1154 (2006). 16608175 10.1021/cr040377d

[b5] Koh C. A., Wisbey R. P., Wu X. P., Westacott R. E. & Soper A. K. Water ordering around methane during hydrate formation. J. Chem. Phys. 113, 6390–6397 (2000).

[b6] Lehmkuhler F. *et al.* The carbon dioxide-water interface at conditions of gas hydrate formation. J. Am. Chem. Soc. 131, 585–589 (2009). 19105749 10.1021/ja806211r

[b7] Moon C., Taylor P. C. & Rodger P. M. Molecular dynamics study of gas hydrate formation. J. Am. Chem. Soc. 125, 4706–4707 (2003). 12696878 10.1021/ja028537v

[b8] Walsh M. R., Koh C. A., Sloan E. D., Sum A. K. & Wu D. T. Microsecond simulations of spontaneous methane hydrate nucleation and growth. Science 326, 1095–1098 (2009). 19815725 10.1126/science.1174010

[b9] English N. J., Johnson J. K. & Tylor C. E. Molecular-dynamics simulations of methane hydrate dissociation. J. Chem. Phys. 123, 244503–244514 (2005). 16396545 10.1063/1.2138697

[b10] Guo G., Zhang Y., Li M. & Wu C. Can the dodecahedral water cluster naturally form in methane aqueous solutions? A molecular dynamics study on the hydrate nucleation mechanisms. J. Chem. Phys. 128, 194504–194511 (2008). 18500877 10.1063/1.2919558

[b11] Jacobson L. C. & Molinero V. A methane-water model for coarse-grained simulations of solutions and clathrate hydrates. J. Phys. Chem. B 114, 7302–7311 (2010). 20462253 10.1021/jp1013576

[b12] Jacobson L. C., Hujo W. & Molinero V. Amorphous precursors in the nucleation of clathrate hydrates. J. Am. Chem. Soc. 132, 11806–11811 (2010). 20669949 10.1021/ja1051445

[b13] Bagherzadeh S. A., Englezos P., Alavi S. & Ripmeester J. A. Influence of hydrated silica surfaces on interfacial water in the presence of clathrate hydrate forming gases. J. Phys. Chem. C 116, 24907–24915 (2012).

[b14] Liang S., Rozmanov D. & Kusalik P. G. Crystal growth simulations of methane hydrates in the presence of silica surfaces. Phys. Chem. Chem. Phys. 13, 19856–19864 (2011). 21879064 10.1039/c1cp21810g

[b15] Bai D., Chen G., Zhang X. & Wang W. Microsecond molecular dynamics simulations of the kinetic pathways of gas hydrate formation from solid surfaces. Langmuir 27, 5961–5967 (2011). 21486061 10.1021/la105088b

[b16] Bai D., Chen G., Zhang X. & Wang W. Nucleation of the CO_2_ hydrate from three-phase contact lines. Langmuir 28, 7730–7736 (2012). 22551251 10.1021/la300647s

[b17] Nakashiki N., Ohsumi T. & Shitashima K. Sequestering of CO_2_ in a deep ocean - fall velocity and dissolution rate of solid CO_2_ in the ocean. CRIEPI Report (EU 91003), Japan, 1991.

[b18] Ozaki M., Sonoda K., Fujioka Y., Tsukamoto O. & Komatsu M. Sending CO_2_ into deep ocean with a hanging pipe from floating platform. Energy Convers. Mgmt. 36, 475–478 (1995).

[b19] Liro C., Adams E. & Herzog H. Modeling the release of CO_2_ in the deep ocean. Energy Convers. Mgmt. 33, 667–674 (1992).

[b20] Haugan P. M. & Drange H. Sequestration of CO_2_ in the deep ocean by shallow injection. Nature 357, 318–320 (1992).

[b21] Ohsumi T. CO_2_ disposal options in the deep sea. Mar. Technol. Soc. J. 29, 58–66 (1995).

[b22] Sloan E. D. Fundamental principles and applications of natural gas hydrates. Nature 426, 353–363 (2003). 14628065 10.1038/nature02135

[b23] Park Y. *et al.* Sequestering carbon dioxide into complex structures of naturally occurring gas hydrates. Proc. Natl. Acad. Sci. USA. 103, 12690–12694 (2006). 16908854 10.1073/pnas.0602251103 PMC1568911

[b24] Koh C. A., Sum A. K. & Sloan E. D. Gas hydrates: unlocking the energy from icy cages. J. Appl. Phys. 106, 061101–061114 (2009).

[b25] Brewer P. G., Friederich C., Peltzer E. T. & Orr F. M. Direct experiments on the ocean disposal of fossil fuel CO_2_. Science 284, 943–945 (1999). 10320370 10.1126/science.284.5416.943

[b26] Rodger P. M., Forester T. R. & Smith W. Simulations of the methane hydrate/methane gas interface near hydrate forming conditions. Fluid Phase Equilib. 116, 326–332 (1996).

[b27] Baez L. A. & Clancy P. Computer simulation of the crystal growth and dissolution of natural gas hydrates. Ann. N.Y. Acad. Sci. 715, 177–186 (1994).

[b28] Moon C., Hawtin R. W. & Rodger P. M. Nucleation and control of clathrate hydrates: insights from simulation. Faraday Discuss. 136, 367–382 (2007). 17955821 10.1039/b618194p

[b29] Matsumoto M., Baba A. & Ohmine I. Topological building blocks of hydrogen bond network in water. J. Chem. Phys. 127, 134504–134512 (2007). 17919034 10.1063/1.2772627

[b30] Jacobson L. C., Hujo W. & Molinero V. Thermodynamic stability and growth of guest-free clathrate hydrates: a low-density crystal phase of water. J. Phys. Chem. B 113, 10298–10307 (2009). 19585976 10.1021/jp903439a

[b31] Page A. J. & Sear R. P. Heterogeneous nucleation in and out of pores. Phys. Rev. Lett. 97, 065701 (2006). 17026175 10.1103/PhysRevLett.97.065701

[b32] Castrillón S. R.-V., Giovambattista N., Aksay I. A. & Debenedetti P. G. Evolution from surface-influenced to bulk-like dynamics in nanoscopically confined water. J. Phys. Chem. B 113, 7973–7976 (2009). 19449830 10.1021/jp9025392

[b33] Argyris D., Tummala N. R. & Striolo A. Molecular structure and dynamics in thin water films at the silica and graphite surfaces. J. Phys. Chem. C 112, 13587–13599 (2008).

[b34] Mosaddeghi H., Alavi S., Kowsari M. H. & Najafi B. Simulations of structural and dynamic anisotropy in nano-confined water between parallel graphite plates. J. Chem. Phys. 137, 184703 (2012). 23163385 10.1063/1.4763984

[b35] Plimpton S. J. Fast parallel algorithms for short-range molecular dynamics. J. Comput. Phys. 117, 1–19 (1995).

[b36] Wiebe R. & Gaddy V. L. The solubility of carbon dioxide in water at various temperatures from 12 to 40° and at pressures to 500 atmospheres. Critical phenomena*. J. Am. Chem. Soc. 62, 815–817 (1940).

[b37] Teng H., Yamasaki A., Chun M.-K. & Lee H. Solubility of liquid CO_2_ in water at temperatures from 278 K to 293 K and pressures from 6.44 MPa to 29.49 MPa and densities of the corresponding aqueous solutions. J. Chem. Thermodynamics 29, 1301–1310 (1997).

[b38] Bamberger A., Sieder G. & Maurer G. High-pressure (vapor+liquid) equilibrium in binary mixtures of (carbon dioxide+water or acetic acid) at temperatures from 313 to 353 K. J. Supercrit. Fluids 17, 97–110 (2000).

[b39] Harris J. G. & Yung K. H. Carbon dioxide’s liquid-vapor coexistence curve and critical properties as predicted by a simple molecular model. J. Phys. Chem. 99, 12021–12024 (1995).

[b40] Jorgensen W. L., Chandrasekhar J., Madura J. D., Impey R. W. & Klein M. L. Comparison of simple potential functions for simulating liquid water. J. Chem. Phys. 79, 926–935 (1983).

[b41] Ryckaert J. P., Ciccotti G. & Berendsen H. J. C. Numerical integration of the Cartesian equations of motion of a system with constraints: molecular dynamics of n-alkanes. J. Comput. Phys. 23, 327–341 (1977).

[b42] Lopes P. E. M., Murashov V., Tazi M., Demchuk E. & MacKerell A. D. Jr. Development of an empirical force field for silica. Application to the quartz-water interface. J. Phys. Chem. B 110, 2782–2792 (2006). 16471886 10.1021/jp055341j PMC2531191

[b43] Hoover W. G. Canonical dynamics: equilibrium phase-space distributions. Phys. Rev. A 31, 1695–1697 (1985). 9895674 10.1103/physreva.31.1695

[b44] Hoover W. G. Constant-pressure equations of motion. Phys. Rev. A 34, 2499–2500 (1986). 9897546 10.1103/physreva.34.2499

[b45] Melchionna S., Ciccotti G. & Holian B. L. Hoover NPT dynamics for systems varying in shape and size. Mol. Phys. 78, 533–544 (1993).

[b46] Allen M. P. & Tildesley D. J. Computer simulation of liquids. (Oxford University Press: Oxford, 2004).

[b47] Schmahl W. W., Swainson I. P., Dove M. T. & Graeme-Barber A. Landau free energy and order parameter behaviour of the phase transition in cristobalite. Z. Kristallogr. 201, 125–145 (1992).

